# Robust Unsupervised Feature Selection Algorithm Based on Fuzzy Anchor Graph

**DOI:** 10.3390/e27080827

**Published:** 2025-08-04

**Authors:** Zhouqing Yan, Ziping Ma, Jinlin Ma, Huirong Li

**Affiliations:** 1 School of Mathematics and Information Science, North Minzu University, Yinchuan 750030, China; 17709239981@163.com; 2 Collaborative Innovation Center for Scientific Computing and Intelligent information Processing, North Minzu University, Yinchuan 750030, China; 3 School of Computer Science and Engineer, North Minzu University, Yinchuan 750030, China; majinlin@nmu.edu.cn; 4 School of Mathematics and Computer Application, Shangluo University, Shangluo 726000, China; lihuirong417@163.com

**Keywords:** unsupervised feature selection, fuzzy weighting, fuzzy graph, orthogonal tri-factorization

## Abstract

Unsupervised feature selection aims to characterize the cluster structure of original features and select the optimal subset without label guidance. However, existing methods overlook fuzzy information in the data, failing to model cluster structures between data effectively, and rely on squared error for data reconstruction, exacerbating noise impact. Therefore, a robust unsupervised feature selection algorithm based on fuzzy anchor graphs (FWFGFS) is proposed. To address the inaccuracies in neighbor assignments, a fuzzy anchor graph learning mechanism is designed. This mechanism models the association between nodes and clusters using fuzzy membership distributions, effectively capturing potential fuzzy neighborhood relationships between nodes and avoiding rigid assignments to specific clusters. This soft cluster assignment mechanism improves clustering accuracy and the robustness of the graph structure while maintaining low computational costs. Additionally, to mitigate the interference of noise in the feature selection process, an adaptive fuzzy weighting mechanism is presented. This mechanism assigns different weights to features based on their contribution to the error, thereby reducing errors caused by redundant features and noise. Orthogonal tri-factorization is applied to the low-dimensional representation matrix. This guarantees that each center represents only one class of features, resulting in more independent cluster centers. Experimental results on 12 public datasets show that FWFGFS improves the average clustering accuracy by 5.68% to 13.79% compared with the state-of-the-art methods.

## 1. Introduction

As a fundamental and efficient technique for reducing computation cost and storage requirements in high-dimensional data, feature selection (FS) has been widely applied in diverse fields such as text classification [1,2], bioinformatics [3], and financial engineering [4]. According to the available label information, FS can be broadly classified into supervised FS algorithms [5], unsupervised feature selection algorithms (UFS) [6,7,8], and semi-supervised feature selection algorithms [9,10,11]. Among these methods, the UFS method has attracted much attention due to its low cost-effectiveness without available labeled data and its robustness against mislabeled data. Importantly, during the feature selection process in UFS, how to empower the identification of hidden structures and patterns in the data with label information is critical for evaluating feature relevance and removing noise and irrelevant features. To address this issue, recent research endeavors have sought to elucidate the characteristics of the feature distribution manifold to successfully improve the efficacy of dimensionality reduction [12,13,14].

In these graph-based UFS algorithms, preserving the local manifold structure of the data can offer topological integrity through a more accurate data representation in the low-dimensional space [15,16,17]. To simultaneously maintain latent structural information in both the data space and feature space, dual graph regularization techniques are introduced into the UFS so that the topology in these spaces can be well maintained [18]. Inevitably, in the process of graph learning, the partitioning of the data based on predefined graphs readily results in sensitivity to variations in the similarity graphs, which enables difficulty in accurately capturing the complex relationships between data points. To alleviate these problems posed by such low-quality similarity graphs, researchers have incorporated adaptive graph learning techniques into UFS algorithms [19]. The inspired intention of these approaches is to transform the neighborhood assignment of the graph into an adaptive process such that optimal neighborhood assignments in each iteration can be investigated to further reduce noise interference. Specifically, to automatically learn the importance of various features, a self-weighting mechanism is constructed in an adaptive graph to guarantee the stability of the manifold structure within both the data and feature spaces [20]. Unlike the self-weighted adaptive graph, a self-representation mechanism is employed to adaptively learn similarity graphs, the aim of which is to learn a projection matrix to guide feature selection [21]. By incorporating adaptive graph learning into latent representation learning, globally interconnected information is explored to clearly reflect relationships between samples, and thus, the adverse effects of noise and redundant information can be mitigated [22]. Recently, with the development of graph fusion techniques, the integration of multiple graph structures has emerged as an effective approach. Especially by learning the fused graph from multiple predefined similarity graphs, it is possible to better leverage the complementary information among the graphs and correct inaccurate similarity values, thereby preserving the local data structure in the projected space [23]. Unfortunately, the issues of excessive graph learning time and the lack of capturing fuzzy relationships between paired sample points in the dimensionality reduction process in feature selection remain unresolved.

Moreover, to alleviate the problem of sensitivity to noise and outliers generated in uncovering manifold information in data in graph learning, it is important to develop different robust unsupervised feature selection (RUFS) methods. A common strategy to enhance robustness is to replace the traditional *F*-norm with the ${𝓁}_{2,1}$-norm or ${𝓁}_{2,1/2}$-norm to constrain the loss term [24]. These norms inherently ignore outliers that exceed a certain threshold, thereby mitigating the impact of noise. Nevertheless, they are still sensitive to small losses in that the limitation of the degree of robustness still exists in these loss functions based on the ${𝓁}_{2,1}$-norm and ${𝓁}_{2,1/2}$-norm. To address this issue, Song et al. proposed an adaptive loss function based on the σ-norm, which was characterized by its ability to adaptively adjust the σ-value according to different situations to minimize the residuals. That is, the σ-norm approximates the ${𝓁}_{2,1}$-norm when dealing with noisy data, whereas it is equivalent to the *F*-norm for smaller losses, such that a significant reduction in the sensitivity of the robust norm to small losses can be provided [25].

In practical applications, assuming consistent importance among features can introduce bias and exacerbate the effects of redundant features and noise on the model [26]. For this reason, Shang et al. proposed an adaptive feature-weighted RUFS method by adaptively assigning weights to features through a weighting matrix to distinguish between the degrees of importance of features, which enhances the focus on discriminative features and increases the likelihood of selecting important features and thus reduces the sensitivity of the model to the outliers [27]. These adaptive feature weighting methods mainly focus on features with relatively higher weight scores, which may lead to the neglect of potentially discriminative features with lower weight scores. In an effort to enhance attention to these secondary features, Wang et al. proposed an exponential weighting learning mechanism that adjusts feature weights using an exponential function, thereby fully exploiting potentially discriminative characteristics [28]. Although this weighting mechanism does enhance the robustness to noise, its ability to detect and eliminate outlier samples remains limited. To tackle this challenge, Huang et al. suggested assigning importance scores to individual samples to ensure that normal samples receive higher scores while noise samples are assigned lower scores [29]. This strategy aids in identifying and excluding outlier points from the data, and thus, the local structure within the robust subspace can be more effectively preserved.

Though the aforementioned UFS models have achieved significant success in dimensionality reduction tasks, challenges still remain in the learning of similarity graphs and weight allocation for data point attributes: (1) Traditional graph regularization methods exhibit high computational complexity during the construction of adjacency graphs and struggle to effectively address the problem of samples being assigned to wrong clusters due to fuzzy relationships between samples. (2) Existing UFS algorithms fail to fully consider the importance of individual features across different samples, which limits the contribution of important features to the samples and leads to an increased error between the ideal and actual values. (3) During feature selection, the quality of the learned low-dimensional representation matrix is often low, thereby resulting in inaccurate clustering structures.

To address the aforementioned issues, this paper proposes a robust unsupervised feature selection algorithm based on a fuzzy anchor graph (FWFGFS). The specific innovations and contributions of this work are as follows:

- This paper proposes a novel fuzzy neighborhood representation mechanism that captures uncertain node–cluster relationships through probabilistic membership distributions. Unlike traditional rigid neighborhood graphs, our approach enables soft cluster assignments while significantly reducing computational complexity from $O(n^{2})$ to O(nl) through efficient anchor approximation. This innovation provides more accurate modeling of real-world data ambiguity while maintaining computational efficiency.
- To effectively handle feature redundancy and noise interference, we develop an adaptive fuzzy weighting system incorporated in the residual term. The system employs a learnable matrix with exponential scaling to dynamically adjust feature importance during optimization. Furthermore, we introduce orthogonal tri-factorization to enforce independence among cluster centers through rigorous orthogonal constraints, which enhances solution stability and prevents degenerate cases common in traditional approaches.
- We present a comprehensive optimization framework with detailed computational complexity analysis. Extensive experiments demonstrate that our method achieves significant speed improvements compared with eight state-of-the-art methods. The proposed approach consistently shows superior clustering performance and stronger noise resistance across various real-world and artificially noised datasets, validating its practical effectiveness.

The remaining content of this paper is structured as follows. Section 2 reviews related algorithms in the field. In Section 3, we present the proposed FWFGFS, detailing the update rules and convergence proof. In Section 4, we validate the superiority of our algorithm through the design of multiple experiments, followed by result analysis. Section 5 concludes the paper with a summary of the research findings and suggestions for future developments.

## 2. Related Work

In this section, some relevant research work in this field is reviewed.

### 2.1. UFS Based on Adaptive Graph and Robust Loss (MFALBS)

As a UFS based on adaptive graph and robust loss, MFALBS performed robust unsupervised feature selection through a joint optimization framework that integrates matrix factorization, adaptive loss, and bi-stochastic graph regularization [30]. Specifically, the adaptive loss term employs a nonlinear weighting scheme to balance the impact of large and small errors, aimed at preserving the fitting capability of the Frobenius norm for smooth data while maintaining the robustness of the ${𝓁}_{2,1}$-norm against outliers. The graph regularization term leverages dynamically optimized bi-stochastic graphs to accurately capture the underlying manifold structure of the data, while the graph learning term ensures the validity and stability of the similarity matrix through constrained optimization. Its objective function is defined as follows:

(1) $$\begin{array}{cc} \; & \min_{W,B,E,S}∥W^{T}X-BE^{T}{∥}_{\sigma }+{\alpha ∥W∥}_{2,1}^{2}+\beta \mathrm{Tr}\left(W^{T}XLX^{T}W\right)+\gamma {∥S-O∥}_{F}^{2}\\ & \;\;s.t.\;E^{T}E=I,B^{T}B=I,E\geq 0,S1_{n}=1_{n},S\geq 0,S^{T}=S,\mathrm{diag}\left(S\right)=0 \end{array}$$

where X is a data matrix, B is a basis matrix, E is a coefficient matrix, W is the feature selection matrix, S denotes the learned similarity matrix, O denotes the original similarity matrix, L is the Laplacian matrix, α,β,γ are constraint parameters, and α,β,γ>0. The adaptive loss function overcomes the limitations of traditional fixed-norm measures by automatically adjusting the error penalty strategy according to data characteristics. Meanwhile, the bi-stochastic graph learning breaks through the constraints of fixed similarity graphs, enabling dynamic optimization of local data structures.

### 2.2. UFS Based on Dual Fuzzy Graph and Orthogonal Basis Clustering (DFGOC)

DFGOC achieves efficient unsupervised feature selection through the synergistic optimization of orthogonal basis clustering and dual space fuzzy graphs [31]. The orthogonal basis clustering module projects the original data into a low-dimensional space via matrix factorization, where the orthogonal constraints imposed on the cluster center matrix F and the clustering indicator matrix G can ensure the independence of cluster centers and the clarity of the clustering structure. The dual space fuzzy graph module is responsible for constructing adaptive similarity graphs in both the data space and feature space, adjusting the similarity weights through the fuzziness parameter *t*, and precisely controlling the number of neighbors for each sample via the ${𝓁}_{2,0}$-norm constraint, effectively addressing the imbalanced neighbor problem inherent in traditional *k*-nearest neighbor graphs. The objective function of this method is defined as

(2) $$\begin{array}{cc} \; & \min_{W,B,E,S,G}∥XW-BE^{T}{∥}_{F}^{2}+\;{\alpha ∥W∥}_{F}^{2}+\frac{\beta }{2}\sum_{i=1}^{d}\sum_{j=1}^{d}{∥{\left(XW\right)}_{i}-{\left(XW\right)}_{j}∥}_{2}^{2}S_{ij}^{t}\\ \; & \;+\frac{\gamma }{2}\sum_{i=1}^{n}\sum_{j=1}^{n}{∥W^{T}X_{i}-W^{T}X_{j}∥}_{2}^{2}G_{ij}^{t}\\ \; & \;s.t.\;E^{T}E=I,B^{T}B=I,B\geq {0,∥W∥}_{2,0}={m,∥S∥}_{2,0}=k,{∥G∥}_{2,0}=k,\\ \; & \;S1_{n}=1_{n},G1_{d}=1_{d},S\geq 0,G\geq 0,t>1 \end{array}$$

where α, β, and γ are the regularization parameters with α,β,γ>0, and B is the basis matrix, E is the coefficient matrix, W is the feature selection matrix, S is the feature graph, G is the data graph, and *t* is the fuzzy coefficient. And ${𝓁}_{2,0}$-norm is defined as ${∥X∥}_{2,0}=\sum_{i=1}^{m}f(∥x_{i}{∥}_{2}\neq 0)$, where f(·) is the indicator function, which equals 1 if the condition is satisfied and 0 otherwise. The adaptive learning mechanism of the fuzzy graphs eliminates the need for manual adjustment of neighbor parameters, significantly reducing model complexity. Meanwhile, the dual space structure preservation ensures the discriminative power of the selected features.

### 2.3. UFS Based on the Exponential Weighting (LLSRFS)

LLSRFS establishes a unified framework by integrating local structure learning and exponentially weighted sparse regression [28]. Mathematically, the objective function employs an exponential weighting mechanism to adjust the feature weight distribution, where the exponent term *q* balances feature importance and prevents a few dominant features from skewing the learning process. Specifically, it optimizes the feature-weighted distance and the sample similarity matrix S to ensure that the sample distribution in the feature subspace aligns with the local geometric structure. Additionally, through constraints involving the Laplacian matrix L and the projection matrix P, the model aligns the low-dimensional embedding space with the category discriminant space, thereby preserving global discriminative information. The objective function expression is as follows:

(3) $$\begin{array}{cc} \; & \min_{W,H,P,F,S}\alpha \left(∥HX^{T}{P-HF∥}_{F}^{2}+\beta {∥W^{-1}P∥}_{F}^{2}\right)\\ \; & \;+\sum_{i=1}^{n}\sum_{j=1}^{n}{∥X_{i}-X_{j}∥}_{ew}^{2}S_{ij}+\gamma S_{ij}^{2}+\mu \mathrm{Tr}\left(F^{T}LF\right)\\ \; & \;s.t.\;F^{T}F=I,1\geq S_{ij}\geq 0,S1_{n}=1_{n},W_{ij}\geq 0 \end{array}$$

where α, β, γ, and μ are the regularization parameters with α,β,γ,μ>0. The strength of this objective function lies in its ability to simultaneously capture both local and global structural information while avoiding distortions in sample distribution and feature structure caused by linear transformations in conventional methods. The exponential weighting mechanism effectively mitigates imbalanced feature weight distributions, allowing more informative features to contribute to model learning, thereby enhancing the robustness and discriminative power of feature selection. Furthermore, by jointly optimizing feature weights, similarity matrices, and projection matrices, the model is dynamically capable of adapting the feature subspace to different data characteristics.

## 3. Proposed Method

**Figure 1:**
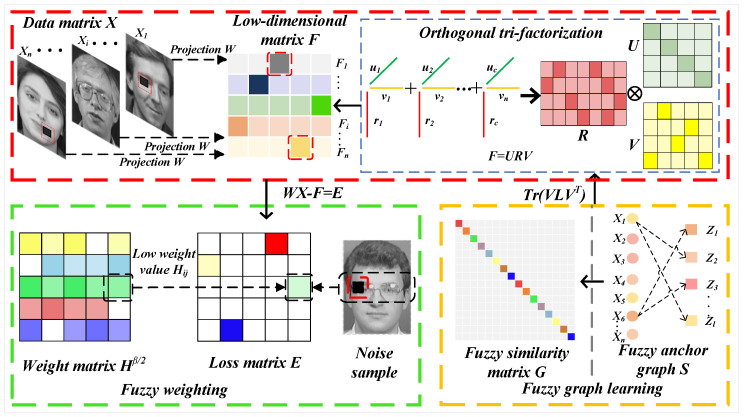
Illustration of the proposed FWFGFS’s feature selection process. In the feature selection process, the sample $x_{i}$ is projected into the low-dimensional space F by the projection matrix W. First, during the adaptive weight learning process, the weight matrix $H^{\beta /2}$ assigns weights to each feature based on the error values, where the red and blue areas represent noise, allocated smaller weights, and the sum of all weights equals 1. Next, a tri-factorization is performed on the low-dimensional matrix F. Finally, a fuzzy anchor graph S is constructed using the sample $x_{i}$ and anchor $z_{j}$, which is then transformed into a fuzzy similarity matrix G. Lastly, a graph regularization constraint is applied to the clustering indicator matrix V.

In this section, we first detail the symbols used in this paper and their corresponding interpretations. In addition, we conduct a problem analysis, then introduce a robust unsupervised feature selection algorithm based on fuzzy anchor graph (FWFGFS), and then give an iterative update scheme of FWFGFS. The framework of FWFGFS is shown in Figure 1.

### 3.1. Notations

Table 1 lists the description on notations.

### 3.2. Problem Formulation

Given a data matrix $X=\left[x_{1},x_{2},\ldots ,x_{n}\right]\in R^{d\times n}$, where *n* denotes the number of samples and *d* represents the number of features for each sample, $x_{ij}$ is defined as the element in the *i*-th row and *j*-th column of the matrix X, and $x_{i}$ is defined as the vector in the *i*-th column of the matrix X.

The key to the UFS algorithm lies in transforming the feature selection problem into a linear regression model. In previous research, a common approach is to use a projection matrix W to map the original data matrix X into a low-dimensional representation matrix V. To minimize the gap between the feature transformation result and the low-dimensional representation matrix F, we introduce a linear regression model that optimizes the feature selection process by minimizing the transformation error. Specifically, the optimization objective is

(4) $$\begin{array}{cc} \; & \min_{W,F}{∥WX-F∥}_{F}^{2}\\ \; & \;s.t.\;WW^{T}=I \end{array}$$

Traditional loss functions typically optimize the discrepancy between the model’s predictions and actual values by minimizing the squared error. However, when the data contains redundant features or noise, this approach may lead to suboptimal performance, as the error between the ideal low-dimensional representation F and the predicted value WX increases with the presence of redundancy or noise. This increase in error contradicts the original goal of minimizing the loss function. Traditional loss functions fail to effectively account for the redundancy and importance of features, which negatively affects the performance of feature selection in complex datasets. To address this issue, this paper proposes an improved loss function based on an adaptive weight matrix H. This strategy is based on the form of the Hadamard product and dynamically adjusts the loss through the weight matrix H according to the error between realistic and ideal values, enabling the model to adaptively mitigate the effects of redundant features and noise in the process of feature selection. The proposed model is thus formulated as

(5) $$\begin{array}{cc} \; & \min_{W,F,H}{∥H^{\frac{1}{2}}⊙\left(WX-F\right)∥}_{F}^{2}\\ \; & \;s.t.\;WW^{T}=I,1\geq H_{ij}\geq 0,\sum_{i=1}^{c}H_{ij}=1 \end{array}$$

During the adaptive optimization of the weight matrix H, when the error between the actual value and the ideal value is large, we assign a smaller weight to that data point to reduce its negative impact on the model. Conversely, if the error is small, a larger weight is assigned to improve its influence in the model learning process. Therefore, the proposed weight matrix H can effectively distinguish between normal data and outliers, thereby preventing outliers from interfering with the model’s learning.

In practical applications, the elements of H are typically binary (0 or 1), which fails to accurately reflect the true weight distribution. To address this, we introduce a fuzzy weighting mechanism with coefficient β(β>1), constraining weights to the [0,1] interval, thus more accurately capturing the continuous variation of the weights.

Specifically, when β approaches 1, the model reduces to a hard-thresholding allocation mechanism, retaining only the features with minimal errors. When β=2, it becomes equivalent to conventional linear weighting. As β increases, the system generates a smooth weight distribution, thereby significantly enhancing model robustness and effectively suppressing noise interference. The model is further optimized as follows:

(6) $$\begin{array}{cc} \; & \min_{W,F,H}{∥H^{\frac{\beta }{2}}⊙\left(WX-F\right)∥}_{F}^{2}\\ \; & \;s.t.\;WW^{T}=I,1\geq H_{ij}\geq 0,\sum_{i=1}^{c}H_{ij}=1 \end{array}$$

The low-dimensional representation matrix F is crucial for realizing the clustering of the data. Inspired by matrix factorization techniques, Miao [32] decomposed F into a clustering center matrix U and a clustering indicator matrix V, applying orthogonality constraints to ensure the structural independence of U and V. However, the dual orthogonal constraints may lead to unreliable solutions because they overly restrict the solution space, forcing both matrices to be strictly orthogonal simultaneously. This rigidity can result in suboptimal clustering structures when the data distribution does not perfectly align with orthogonal assumptions, especially in noisy or complex datasets.

To resolve this issue, we introduce an auxiliary matrix R, which facilitates a tri-factorization of F, namely F=URV. The tri-factorization provides additional flexibility by decoupling the orthogonal constraints through R, allowing U and V to adapt more freely to the underlying data structure. Specifically, R acts as a transformation matrix that

- Relaxes the strict orthogonality requirements.
- Preserves the independence of cluster centers.
- Maintains the discriminative power of features.

This approach effectively prevents the emergence of unreliable solutions caused by overly rigid constraints and enhances the robustness of the model. The model is further optimized as

(7) $$\begin{array}{cc} \; & \min_{W,V,H,U,R}{∥H^{\frac{\beta }{2}}⊙\left(WX-URV\right)∥}_{F}^{2}\\ \; & \;s.t.\;WW^{T}=I,UU^{T}=I,VV^{T}=I,1\geq H_{ij}\geq 0,\sum_{i=1}^{e}H_{ij}=1 \end{array}$$

The manifold structure of the data plays an important role in revealing the distribution characteristics of the data and improving the dimensionality reduction performance. However, traditional neighborhood graphs rely on hard assignments (e.g., k-nearest neighbors), which cannot effectively simulate the uncertainty and ambiguity inherent in real-world data. For instance, samples near cluster boundaries or in noisy regions may not belong strictly to a single cluster, leading to incorrect neighbor pairing in the graph.

To overcome this limitation, this paper proposes a fuzzy anchor graph regularization method, which generalizes traditional anchor graphs by incorporating fuzzy set theory. Unlike hard clustering-based graphs, the fuzzy anchor graph assigns each sample a membership degree to multiple anchors, reflecting the likelihood of affiliation. This soft assignment captures the inherent uncertainty in data relationships, making the graph more robust to noise and outliers.

In this method, we employ soft clustering to compute fuzzy memberships, allowing samples to have varying degrees of affiliation across multiple anchors rather than being restricted to a single cluster. This flexibility mitigates the risk of incorrect cluster assignments and enhances the graph’s adaptability to complex data distributions. The objective function for learning the fuzzy anchor graph *S* is defined as

(8) $$\begin{array}{cc} \; & \min_{s}\sum_{i=1}^{n}\sum_{j=1}^{l}{∥x_{i}-z_{j}∥}_{2}^{2}s_{ij}^{\gamma }\\ \; & \;s.t.\;1\geq s_{ij}\geq 0,s_{i}^{T}1_{n}=1_{n} \end{array}$$

where $Z=\left[z_{1},z_{2},\ldots ,z_{l}\right]\in R^{d\times l}$ represents the anchor matrix, $z_{i}$ is the *i*-th anchor, *l* is the number of anchors, γ is the fuzzy coefficient, and S is the fuzzy anchor graph. (8) introduces the Lagrange multiplier $\theta_{i}$, which can be converted into a Lagrangian function about S:

(9) $$\begin{array}{c} L\left(s_{ij},\theta_{i}\right)=\sum_{i=1}^{n}\sum_{j=1}^{l}{∥x_{i}-z_{j}∥}_{2}^{2}s_{ij}^{\gamma }-\sum_{i=1}^{n}\theta_{i}\left(\sum_{j=1}^{l}s_{ij}-1\right) \end{array}$$

Taking the derivative of $\theta_{i}$ and $s_{ij}$ in L, respectively, and setting their derivatives to zero,

(10) $$\begin{array}{c} \frac{\partial L(s_{ij},\theta_{i})}{\partial s_{ij}}=\gamma {∥x_{i}-z_{j}∥}_{2}^{2}s_{ij}^{\gamma -1}-\theta_{i}=0, \end{array}$$

(11) $$\begin{array}{c} \frac{\partial L(s_{ij},\theta_{i})}{\partial \theta_{i}}=\sum_{j=1}^{l}s_{ij}-1=0. \end{array}$$

From (10), we obtain

(12) $$s_{ij}={\left(\frac{\theta_{i}}{\gamma ∥x_{i}-z_{j}{∥}_{2}^{2}}\right)}^{\;\;1/(\gamma -1)}$$

According to (12),we obtain the following:

(13) $$\sum_{j=1}^{l}s_{ij}=\sum_{j=1}^{l}{\left(\frac{\theta_{i}}{\gamma ∥x_{i}-z_{j}{∥}_{2}^{2}}\right)}^{\;\;1/(\gamma -1)}=1$$

So there are

(14) $${\left(\frac{\theta_{i}}{\gamma }\right)}^{\;\;1/(\gamma -1)}={\left[\sum_{j=1}^{l}{\left(\frac{1}{∥x_{i}-z_{j}{∥}_{2}^{2}}\right)}^{\;\;1/(\gamma -1)}\right]}^{-1}$$

The final update formula for $s_{ij}$ is

(15) $$s_{ij}=\left\{\begin{array}{cc} \frac{∥x_{i}-z_{j}{∥}_{2}^{-2/(\gamma -1)}}{\sum_{k=1}^{l}{∥x_{i}-z_{k}∥}_{2}^{-2/(\gamma -1)}}, & \mathrm{if}\;z_{j}\in \mathrm{KNN}\left(x_{i}\right)\\ 0, & \mathrm{otherwise} \end{array}\right.$$

Membership $s_{ij}$ decays with distance $∥x_{i}-z_{j}{∥}_{2}$, but the decay rate is controlled by γ. To improve computational efficiency, $s_{ij}$ is non-zero only for the *k*-nearest anchors of $x_{i}$, balancing local structure preservation and scalability.

The fuzzy Laplacian encodes uncertain relationships more accurately than binary graphs, improving robustness in downstream tasks like clustering or dimensionality reduction. This paper performs fuzzy neighborhood manifold learning based on an anchor graph on the clustering indicator matrix. The objective function is defined as

(16) $$\min_{V}Tr\left(VLV^{T}\right)$$

where *L* represents the Laplacian matrix, defined as L=D−G. Here, G is the fuzzy similarity matrix between data points, and D is the diagonal matrix with elements $d_{ii}=\sum_{j=1}^{n}g_{ij}$.

The fuzzy similarity matrix G is derived from the fuzzy anchor graph S:

(17) $$G=SO^{-1}S^{T}$$

where O is a diagonal matrix with elements $o_{jj}=\sum_{i=1}^{n}s_{ij}$.

The complete objective function is

(18) $$\begin{array}{cc} \; & \min_{W,V,H,U,R}{∥H^{\frac{\beta }{2}}⊙\left(WX-URV\right)∥}_{F}^{2}+\alpha \mathrm{Tr}\left(VLV^{T}\right)\\ \; & \;s.t.\;WW^{T}=I,UU^{T}=I,VV^{T}=I,1\geq H_{ij}\geq 0,\sum_{i=1}^{c}H_{ij}=1 \end{array}$$

Through the adaptive fuzzy weight matrix H, our method dynamically adjusts sample and feature weights to effectively reduce noise interference. The orthogonal tri-factorization F=URV enhances stability compared with traditional dual orthogonal constraints. Furthermore, the fuzzy anchor graph regularization optimizes the data neighborhood structure, improving dimensionality reduction performance.

### 3.3. Optimization Procedure

In this section, we detail how to solve the optimization problem of (18). We split the optimization problem in (18) into five independent subproblems to solve W, V, U, R, and  H, respectively. We devised an alternating iterative algorithm to solve this optimization problem.

#### 3.3.1. Update *H*

The optimization subproblem for H can be reformulated as

(19) $$\begin{array}{cc} \; & \min_{H}{∥H^{\frac{\beta }{2}}⊙\left(WX-URV\right)∥}_{F}^{2}\\ \; & \;s.t.\;1\geq H_{ij}\geq 0,\;\sum_{i=1}^{c}H_{ij}=1 \end{array}$$

Let E=WX−URV; we rewrite (19) as

(20) $$\begin{array}{cc} \; & \min_{H}\sum_{i=1}^{c}\sum_{j=1}^{n}H_{ij}^{\beta }E_{ij}^{2}\\ \; & \;s.t.\;1\geq H_{ij}\geq 0,\sum_{i=1}^{c}H_{ij}=1 \end{array}$$

The Lagrangian function is

(21) $$J_{1}=\sum_{i=1}^{c}\sum_{j=1}^{n}H_{ij}^{\beta }E_{ij}^{2}-\sum_{j=1}^{n}\tau_{j}\left(\sum_{i=1}^{c}H_{ij}-1\right)$$

where $\tau_{j}$ are Lagrange multipliers. Setting derivatives to zero,

(22) $$\frac{\partial J_{1}}{\partial \tau_{j}}=\sum_{i=1}^{c}H_{ij}-1=0$$

(23) $$\frac{\partial J_{1}}{\partial H_{ij}}=\beta H_{ij}^{\beta -1}E_{ij}^{2}-\tau_{j}=0$$

From (23), we obtain

(24) $$H_{ij}={\left(\frac{\tau_{j}}{\beta E_{ij}^{2}}\right)}^{\;\;1/(\beta -1)}$$

and from (22) and (24),

(25) $$\sum_{i=1}^{c}H_{ij}=\sum_{i=1}^{c}{\left(\frac{\tau_{j}}{\beta E_{ij}^{2}}\right)}^{\;\;1/(\beta -1)}=1$$

From the previous derivation, we obtain

(26) $${\left(\frac{\tau_{j}}{\beta }\right)}^{\;\;1/(\beta -1)}={\left[\sum_{i=1}^{c}{\left(\frac{1}{E_{ij}^{2}}\right)}^{\;\;1/(\beta -1)}\right]}^{-1}$$

The final update rule for $H_{ij}$ is

(27) $$H_{ij}=\frac{E_{ij}^{-2/(\beta -1)}}{\sum_{k=1}^{c}E_{kj}^{-2/(\beta -1)}},$$

#### 3.3.2. Update Rules for *V*, *U*, *R*, and *W*

The Lagrangian function for (18) is

(28) $$\begin{array}{cc} J= & Tr\left({\left(H^{\beta /2}⊙\left(WX-URV\right)\right)}^{T}\left(H^{\beta /2}⊙\left(WX-URV\right)\right)\right)+\alpha Tr\left(VLV^{T}\right)\\ \; & +Tr\left(\left(VV^{T}-I\right){\left(VV^{T}-I\right)}^{T}\right)+Tr\left(\left(WW^{T}-I\right){\left(WW^{T}-I\right)}^{T}\right)\\ \; & +Tr\left(\left(U^{T}U-I\right){\left(U^{T}U-I\right)}^{T}\right)+Tr\left(\mu V\right)+Tr\left(\rho U\right)+Tr\left(\varphi R\right)+Tr\left(\omega W\right) \end{array}$$

The partial derivatives yield

(29) $$\begin{array}{c} \frac{\partial J}{\partial V}=2R^{T}U^{T}\left(H^{\beta }⊙\left(URV\right)\right)-2R^{T}U^{T}\left(H^{\beta }⊙\left(WX\right)\right)+2\alpha VL+4VV^{T}V-4V+\mu \end{array}$$

(30) $$\begin{array}{c} \frac{\partial J}{\partial U}=2\left(H^{\beta }⊙\left(URV\right)\right)V^{T}R^{T}-2\left(H^{\beta }⊙\left(WX\right)\right)V^{T}R^{T}+4UU^{T}U-4U+\rho \end{array}$$

(31) $$\begin{array}{c} \frac{\partial J}{\partial R}=2U^{T}\left(H^{\beta }⊙\left(URV\right)\right)V^{T}-2U^{T}\left(H^{\beta }⊙\left(WX\right)\right)V^{T}+\varphi \end{array}$$

(32) $$\begin{array}{c} \frac{\partial J}{\partial W}=2\left(H^{\beta }⊙\left(WX\right)\right)X^{T}-2\left(H^{\beta }⊙\left(URV\right)\right)X^{T}+4WW^{T}W-4W+\omega \end{array}$$

The KKT conditions lead to the following iterative update rules:

(33) $$V_{ij}\leftarrow V_{ij}\frac{{\left[R^{T}U^{T}\left(H^{\beta }⊙\left(WX\right)\right)+\alpha VG+2V\right]}_{ij}}{{\left[R^{T}U^{T}\left(H^{\beta }⊙\left(URV\right)\right)+2VV^{T}V+\alpha VD\right]}_{ij}}$$

(34) $$U_{ij}\leftarrow U_{ij}\frac{{\left[\left(H^{\beta }⊙\left(WX\right)\right)V^{T}R^{T}+2U\right]}_{ij}}{{\left[\left(H^{\beta }⊙\left(URV\right)\right)V^{T}R^{T}+2UU^{T}U\right]}_{ij}}$$

(35) $$R_{ij}\leftarrow R_{ij}\frac{{\left[U^{T}\left(H^{\beta }⊙\left(WX\right)\right)V^{T}\right]}_{ij}}{{\left[U^{T}\left(H^{\beta }⊙\left(URV\right)\right)V^{T}\right]}_{ij}}$$

(36) $$W_{ij}\leftarrow W_{ij}\frac{{\left[\left(H^{\beta }⊙\left(URV\right)\right)X^{T}+2W\right]}_{ij}}{{\left[\left(H^{\beta }⊙\left(WX\right)\right)X^{T}+2WW^{T}W\right]}_{ij}}$$

The workflow of FWFGFS is illustrated in Algorithm 1.
**Algorithm 1:** Robust unsupervised feature selection based on the fuzzy anchor graph.**Input**: Data matrix $X\in R^{d\times n}$; the number of clusters *c*; the number of neighbors *k*; parameters α,β,γ; the maximum number of iterations *T*; the number of feature selection *p*. **Output**: Feature subset $X_{\mathrm{new}}\in R^{c\times n}$ **Initialization**: Matrix H,W,U,R, and V; the iteration times t=0; Laplacian matrix L. **While** not converged or t≤T   Update H by using (27);   Update V by using (33);   Update U by using (34);   Update R by using (35);   Update W by using (36);   Update *t* by: t=t+1,t≤T;
**EndWhile**
Calculate the evaluation scores for all the features according to $∥W(c,i)∥,\;(i=1,2,\ldots ,d)$. In descending order, we select the top *p* features to form a feature subset $X_{\mathrm{new}}\in R^{c\times n}$.


### 3.4. Complexity Analysis

The overall computational complexity of the FWFGFS consists of initialization and iterative optimization phases. The initialization phase requires O(nl) operations for constructing the fuzzy anchor graph, $O(n^{2}l)$ for computing the fuzzy similarity matrix through pairwise sample comparisons across *l* anchors, and $O(n^{2})$ for deriving the Laplacian matrix.

During the iterative phase, each update cycle demonstrates polynomial complexity:

- Weight matrix H updates scale as O(cn).
- Matrix V optimization involves $O(c^{2}n+cnd+c^{3}+cn)$.
- Centroid matrix U refinement shows $O(c^{3}+c^{2}n)$ complexity.
- Rotation matrix R adjustment requires $O(c^{2}n+cnd+c^{3})$ operations.
- Projection matrix W learning contributes $O(c^{2}n+c^{2}d+cnd+c^{3})$ complexity.

After *T* iterations, the total complexity aggregates to $O\left(T\times \left(c^{3}+c^{2}n+cnd+c^{2}d+n^{2}+cn\right)\right)+O\left(n^{2}l+n^{2}+nl\right)$.

## 4. Experiment Results and Analysis

To validate the superiority of FWFGFS, a series of experiments, including a clustering experiment, ablation experiment, noise test, feature selection effectiveness evaluation, convergence analysis, parameter sensitivity analysis, intuitive validation of fuzzy anchor graph structure, t-SNE visualization experiment, calculation time analysis, and analysis of parameters of fuzzy anchor graph, are designed. All experiments are conducted in the following environment: an i7-12700 2.10 GHz CPU with 64 GB of RAM running on a Windows system, using MATLAB R2023a as the software platform.

### 4.1. Clustering Experiments

To validate the superiority of the proposed FWFGFS method, this section conducts comparative clustering experiments using the K-means algorithm against eight state-of-the-art approaches across twelve publicly available datasets.

#### 4.1.1. Experiment Preparation

This study utilizes 12 publicly available datasets, encompassing both image and text data, to validate the performance of the proposed algorithm. The image datasets include ORL, YaleB, imm40, Jaffe, Jaffe50, Yale64, UMIST_fac, warpPIE10P, and orlraws10P, in which the ALLAML dataset is a well-known gene expression dataset, and the text datasets contain RELATHE and k1a, providing a rich sample for feature selection research. The specific information is listed in Table 2.

In this study, eight of the recent UFS algorithms are selected as comparative methods, a detailed overview of which is provided below:

- SUP [33]: This method combined feature selection and extraction by employing sparse projection matrices and purification matrices to effectively remove redundant information.
- UFS^2^ [34]: A unified learning approach is employed, embedding a binary feature selection vector into K-means, which allows for precise feature selection and avoids the suboptimal issues of traditional methods that select features before clustering.
- VCSDFS [35]: As an unsupervised feature selection method based on variance distance, it excludes features that differ significantly from the original set and selects a more discriminative subset.
- DHBWSL [36]: This method improves feature selection performance by leveraging dual high-order graph learning and Boolean weight adaptive learning to capture the local geometric structures in both data and feature spaces.
- UDS^2^FS [37]: To seek the discriminative subspace, through maximizing interclass divergence and minimizing within-class divergence, UDS^2^FS utilized soft label information to guide this process.
- LRPFS [38]: This method assigns attribute scores to samples through latent learning to enhance the ability to discriminate against outliers.
- RAFG [39]: By employing an adaptive graph to capture clustering distributions and applying ${𝓁}_{2,1}$ norm constraints and ${𝓁}_{2,p}$ norm regularization, noise and irrelevant features are able to be reduced.
- BGLR [40]: Addressing feature redundancy and computational complexity by selecting anchors based on sample variance, an adaptive anchor graph was constructed with ${𝓁}_{2,0}$ norm constraints, applying to provide a discriminative feature subset with low redundancy regularization.

In the experiments, the search range for the balancing parameter α in FWFGFS is set to $\left\{10^{-4},10^{-3},10^{-2},10^{-1},1,10^{1},10^{2},10^{3},10^{4}\right\}$. The range for the fuzzy coefficients β and γ is set to {1.5,2,2.5,3,3.5,4,4.5,5,5.5} and {1.2,1.3,1.4,1.5,1.6,1.7,1.8,1.9,2}, respectively. For the comparative methods, the parameters are uniformly set to $\left\{10^{-4},10^{-3},10^{-2},10^{-1},1,10^{1},10^{2},10^{3},10^{4}\right\}$. The number of selected features is uniformly set to {20,30,40,50,60,70,80,90,100}. In addition, the number of neighbors *k* in the neighbor graph is set to 5. In the clustering experiment, the maximum number of iterations is set to 30, and the iteration is terminated prematurely when the objective function value Obj(t) meets $|Obj\left(t\right)-Obj\left(t-1\right)|/Obj\left(t-1\right)<10^{-6}$. To ensure the stability and reliability of the experimental results, all algorithms are repeated 20 times, and the average values are computed as the final results.

#### 4.1.2. Clustering Results and Analysis

In this section, to evaluate the effectiveness of the proposed FWFGFS, clustering experiments are conducted on 12 datasets compared with eight relevant methods, and the experimental results are presented in Table 3 and Table 4, where the optimal outcomes for each dataset are highlighted in bold.

FWFGFS consistently outperforms all comparative algorithms in accuracy across 12 public datasets. In terms of ACC, FWFGFS achieves an average clustering accuracy improvement of 11.63%, 12.56%, 13.79%, 6.98%, 11.38%, 9.72%, 5.68%, and 10.98% over SUP, UFS^2^, VCSDFS, DHBWSL, UDS^2^FS, LRPFS, RAFG, and BGLR, respectively. This significant advantage primarily stems from the adaptive fuzzy weighting mechanism, which dynamically adjusts feature weights to reduce noise impact while preserving local structural information through effective regularization of the fuzzy anchor graph.

Although VCSDFS, LRPFS, and UDS^2^FS demonstrate effectiveness in certain aspects of feature selection, they face challenges in handling complex structured data. For instance, LRPFS achieves a maximum accuracy of 60.29% on the imm40 dataset, benefiting from its enhanced ability to identify outliers through the allocation of latent attribute scores to samples. However, its performance on the warpPIE10P dataset, with an ACC of merely 33.88%, falls short of the 37.97% achieved by UDS^2^FS. In contrast, the UDS^2^FS, which employs soft labels to guide feature selection, demonstrates relatively superior performance, with average cluster accuracy surpassing UFS^2^ and VCSDFS by 1.18% and 2.39%, respectively.

Furthermore, DHBWSL, RAFG, and BGLR exhibit moderate efficacy in capturing local structures through graph learning techniques; however, they still face challenges when addressing complex structural data. For example, DHBWSL records ACC values of 79.92% and 42.66% on the Jaffe50 and k1a datasets, both of which are lower than the 81.50% and 59.35% achieved by LRPFS. This discrepancy can be attributed to the fact that, while high-order graph learning can effectively capture local structures, its capability to recognize noise remains limited. RAFG retains local manifold structures through adaptive graph learning, achieving suboptimal ACC results of 51.24% and 84.64% on the UMIST_fac and Jaffe50 datasets, respectively. In contrast, BGLR exhibits relatively lower performance, with an average clustering accuracy of 54.82%, which is substantially lower than that of DHBWSL and RAFG.

### 4.2. Noise Test

**Figure 2:**
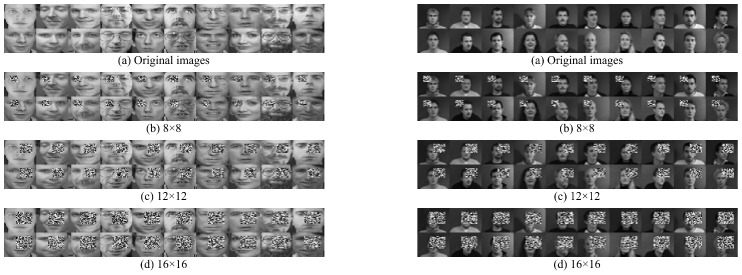
Samples from the (**left**) ORL and (**right**) imm40 datasets with noise of different sizes.

To assess the robustness of FWFGFS, noise experiments are performed on six noisy datasets. We randomly selected 20 samples from the ORL and imm40 datasets, then added occlusion noise (random grayscale blocks) with three sizes: 8 × 8, 12 × 12, and 16 × 16 pixels. The details of the noisy dataset are shown in Table 5 and Figure 2.

From the clustering results depicted in Table 6 and Table 7, where the optimal outcome for each dataset is represented in bold, we can draw the following conclusions:

On these noisy datasets from ORL and imm40, FWFGFS achieves the optimal ACC and NMI results, demonstrating its strong robustness. Specifically, in terms of ACC, compared with the SUP, UFS^2^, VCSDFS, DHBWSL, UDS^2^FS, LRPFS, RAFG, and BGLR algorithms, FWFGFS improves the average clustering accuracy by 5.94%, 14.46%, 16.64%, 10.73%, 10.54%, 10.58%, 4.58%, and 9.43%, respectively. The reason for this significant performance enhancement may lie in the adaptive fuzzy weighting mechanism, which dynamically allocates different weights according to the degree of deviant features, thereby further discriminating these noisy samples. That is, this effectively mitigates the interference from outlier samples and highlights the contribution of key features to the optimization process.

In contrast, though RAFG and SUP demonstrate some robustness to noisy datasets to an extent, with average ACC values of 57.18% and 55.81%, respectively, their performance significantly deteriorates in complex noisy scenarios such as the imm40_16 dataset. This robustness stems from the adaptive graph optimization techniques in RAFG, which perform neighbor selection to effectively eliminate the influence of noisy datasets, and the characteristics of the purification matrices in SUP, which enable the selection of a more robust subset of features. Meanwhile, VCSDFS and UFS^2^ exhibit poor performance under noise interference, with average ACC values of 45.11% and 47.30% in the noisy datasets, which are substantially lower than the other comparison methods. The reasons for these are that both the variance distance metric method of VCSDFS and the binary feature selection vector in UFS^2^ are highly sensitive to noise.

### 4.3. Ablation Study

**Figure 3:**
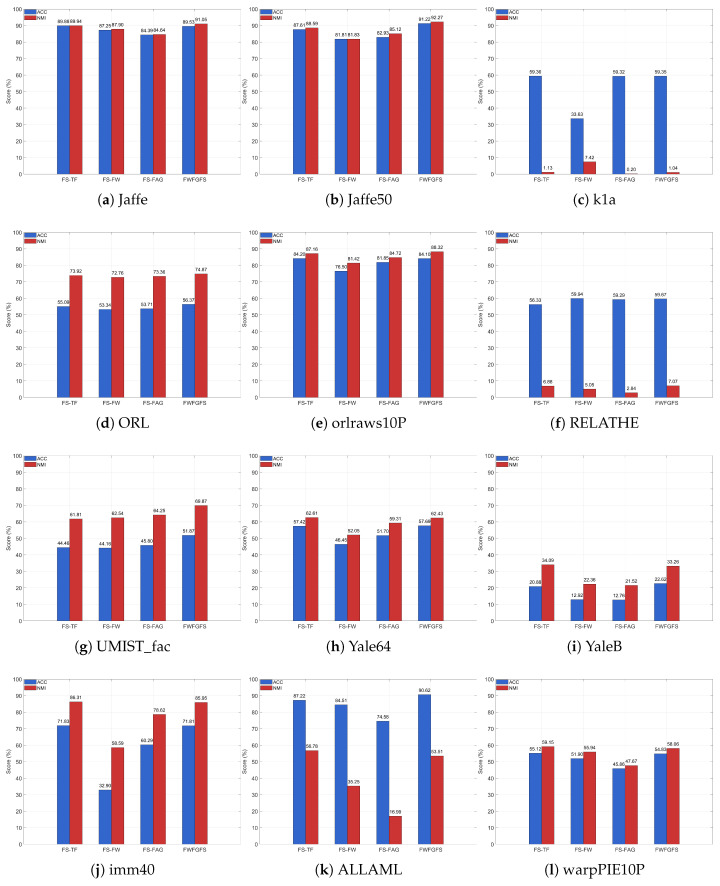
Clustering results of the datasets in the ablation experiment.

To investigate the impact of each component of the proposed method on its performance, we conduct ablation experiments on the ORL, YaleB, Jaffe, orlraws10P, RELATHE, Jaffe50, Yale64, UMIST_fac, warpPIE10P, imm40, ALLAML, and k1a datasets. In these experiments, these variant models, FS-FW, FS-TF, and FS-FAG, are derived from FWFGFS by removing specific components, namely the fuzzy weighting mechanism, orthogonal tri-factorization, and fuzzy graph regularization (see Figure 3).

From an overall performance perspective, the proposed FWFGFS method demonstrates significant advantages in both clustering ACC and NMI metrics. Calculating the average performance across all datasets, FWFGFS achieves an average ACC of 65.81%, outperforming FS-TF (64.12%), FS-FW (55.44%), and FS-FAG (59.37%). Similarly, its average NMI reaches 59.80%, surpassing FS-TF (59.03%), FS-FW (51.76%), and FS-FAG (51.52%). These results indicate that by integrating the fuzzy weighting mechanism, orthogonal tri-factorization, and fuzzy graph regularization, our model can more stably enhance feature selection performance, particularly excelling in data structure preservation and clustering consistency.

Further analysis of the ablation models reveals notable differences in the impact of each component. For the YaleB and k1a datasets, removing the fuzzy weighting mechanism (FS-FW) leads to sharp declines in ACC by 9.7% and 25.72%, respectively, demonstrating that this mechanism significantly improves the model’s robustness to noise and sample imbalance through adaptive weight allocation. On datasets with distinct manifold structures, such as RELATHE and UMIST_fac, removing fuzzy graph regularization (FS-FAG) results in NMI reductions of 4.23% and 5.62%, respectively, confirming that this component effectively captures local geometric relationships in the data via fuzzy similarity matrices. These findings fully substantiate that the complete model achieves optimal performance across various types of data.

### 4.4. Convergence Analysis

**Figure 4:**
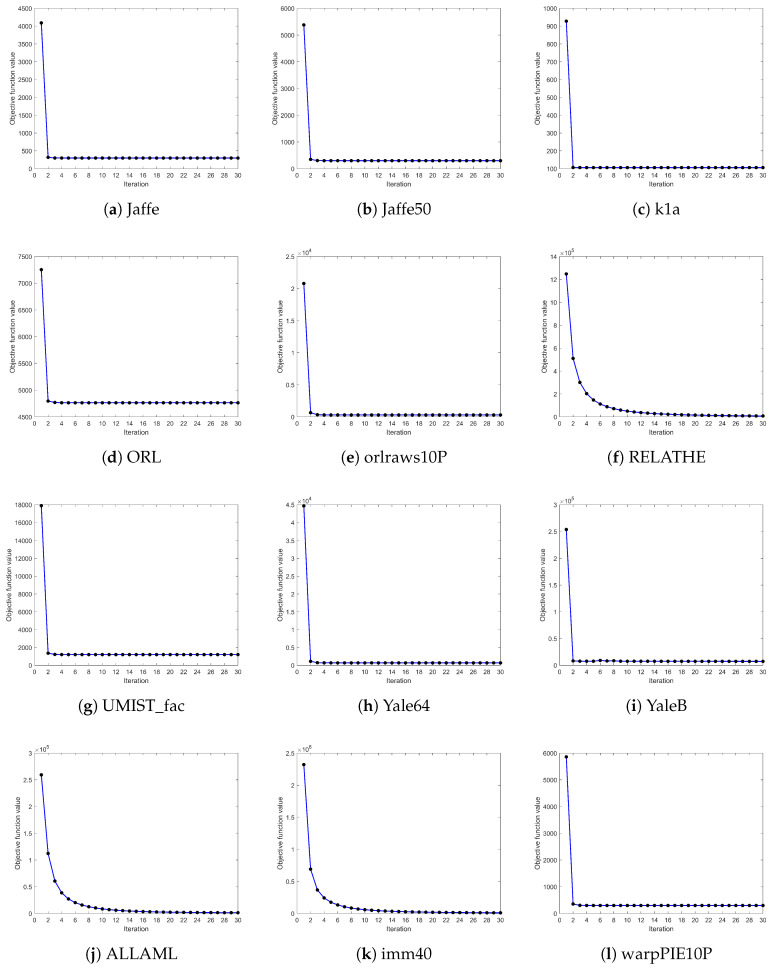
Convergence curve.

In this section, we investigate the convergence behavior of FWFGFS by analyzing the changes in the objective function value after each iteration. Figure 4 illustrates the convergence curves of the proposed algorithm on 12 datasets. In the preliminary phase, the value of the objective function decreases rapidly, signifying that FWFGFS demonstrates the capacity to efficiently converge toward the optimal solution. As the iterations progress, the objective function value gradually stabilizes, reflecting that FWFGFS has reached a steady state. In subsequent iterations, the objective function value generally maintains a non-increasing trend, with the fluctuation amplitude progressively diminishing towards zero. This implies that FWFGFS exhibits good stability and convergence efficiency in effectively finding the local optimal solution.

### 4.5. Parameter Sensitivity Analysis

**Figure 5:**
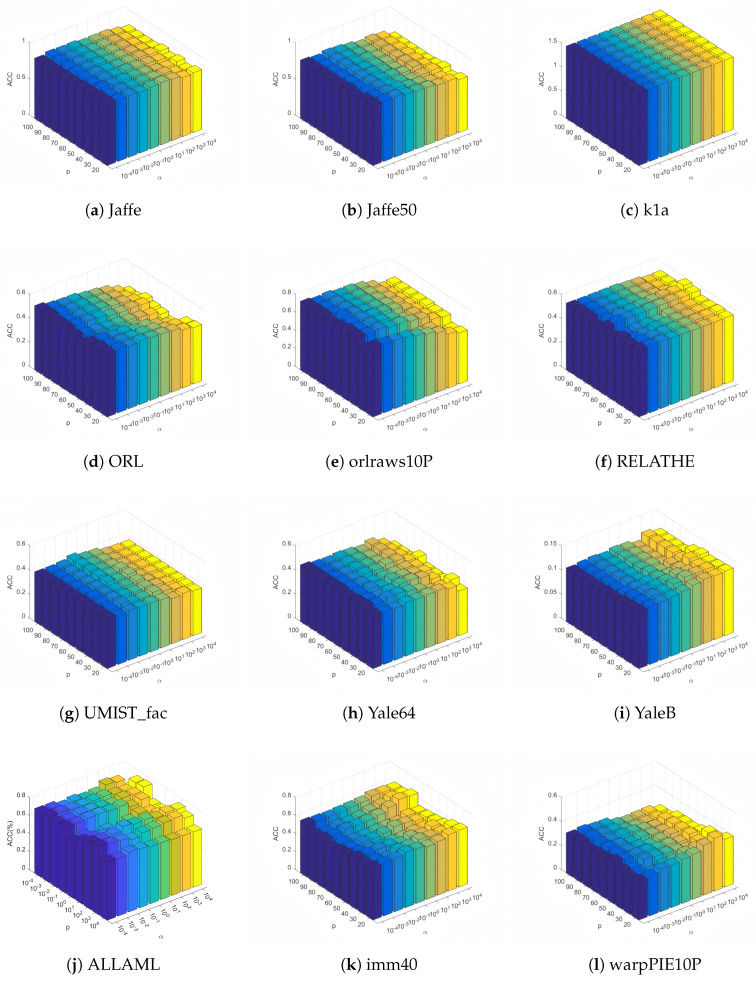
Clustering accuracy for different values of the parameter α and the number of feature selection *p* on test datasets.

**Figure 6:**
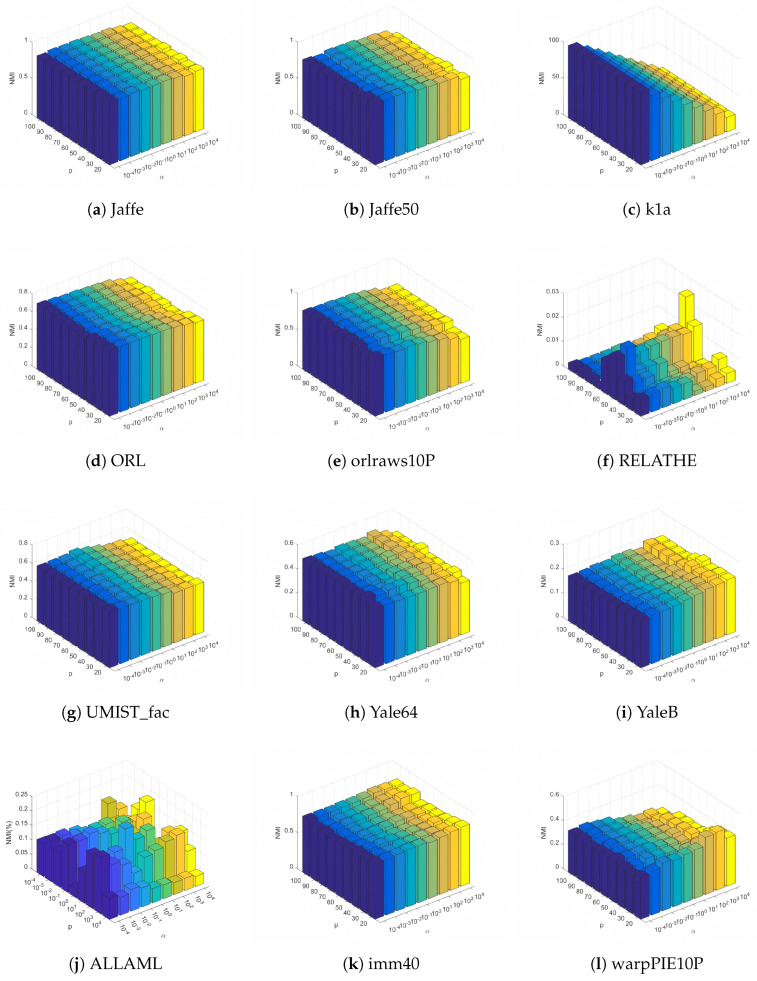
Normalized mutual information for different values of the parameter α and the number of feature selection *p* on test datasets.

In this subsection, we conduct a sensitivity analysis of the balance parameter α and the number of selected features *p* in the objective function on 12 datasets with fuzzy coefficients β and γ set to 1.5. Moreover, the value ranges for parameter α and the number of selected features *p* are in the range of $\left\{10^{-4},10^{-3},10^{-2},10^{-1},1,10^{1},10^{2},10^{3},10^{4}\right\}$ and {20,30,40,50,60,70,80,90,100}, respectively. The ACC and NMI values of FWFGFS under different combinations of α and *p* are displayed in the form of three-dimensional histograms, as shown in Figure 5 and Figure 6.

From these histograms, it can be observed that on most of the datasets (such as YaleB, Jaffe50, Jaffe, Yale64, orlraws10P, ORL, and UMIST_fac), the ACC and NMI values of FWFGFS exhibit relatively small changes with variations in α and *p*. Though some small fluctuations in ACC and NMI are observed on the ALLAML, imm40, and warpPIE10P datasets, FWFGFS demonstrates strong stability and robustness on most of the datasets, especially on the Jaffe50 and Jaffe datasets, where the variations in ACC and NMI are minimal. In conclusion, for FWFGFS, the overall performance remains very stable, and the optimal parameter combination for a given dataset can be determined via grid search.

### 4.6. Intuitive Validation of Fuzzy Anchor Graph Structure

In this subsection, to validate the efficacy of the proposed fuzzy graph learning in capturing the local manifold structure of the data, a visualization experiment is conducted on the ORL_16 dataset. This experiment involves a comparative analysis with several benchmark algorithms: SUP, which employs a traditional predefined similarity graph; RAFG, which utilizes an adaptive similarity graph; DHBWSL, based on a high-order fusion graph; and FWFGFS, which leverages a fuzzy anchor graph.

**Figure 7:**
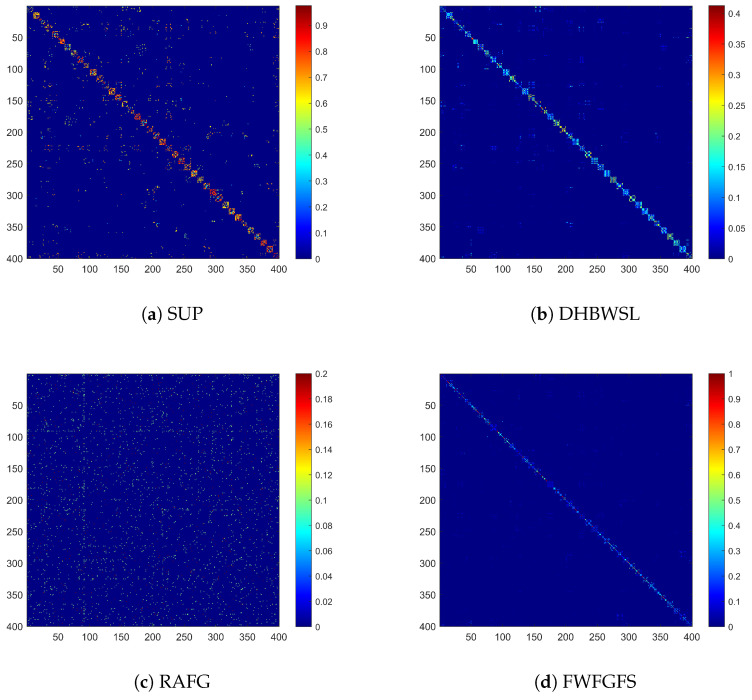
Intuitive validation of the graph on the ORL_16 dataset.

The experimental results presented in Figure 7 distinctly demonstrate that the graph structure generated by FWFGFS effectively captures a consistent and robust structure that accurately reflects the intricate relationships inherent in the data, thereby establishing itself as the most coherent among the evaluated approaches. These structures gleaned from the similarity graph of RAFG are marred by considerable noise. This is largely attributable to its similarity graph learning measure, which inadequately removes noise and fails to accurately delineate the underlying data structure. Although DHBWSL exhibits a degree of advancement compared with RAFG, the graphs they produced remain notably ambiguous and exhibit some degree of noise interference. These results further confirm the effectiveness of the fuzzy anchor graph employed in our proposed method, highlighting its capacity to enhance the quality of graph structures.

### 4.7. Effectiveness Experiment of Feature Selection

**Figure 8:**
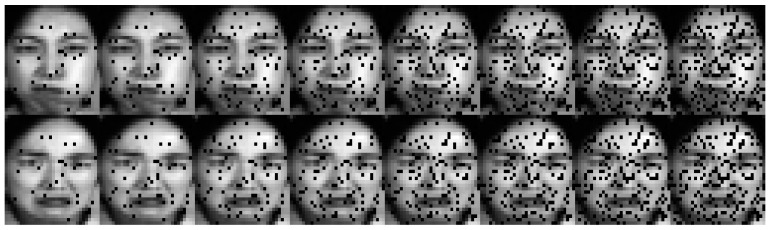
Results of two Jaffe50 samples with different numbers of selected features.

In this work, to validate the discriminative power of the selected features, we visualized the features selected by the proposed method. Especially, two samples are randomly selected from the Jaffe50 dataset, and then feature selection is performed by the proposed method with the number of selected features set to 30, 50, 80, 100, 130, 150, 180, and 200. Figure 8 displays the corresponding images with different numbers of selected features marked by black pixels.

It can be seen from Figure 8 that when the number of selected features is set to 30, the selected features mainly concentrate on the nose, eyebrows, and mouth regions. As the number of selected features increases to 50, regions such as the forehead, eyebrows, mouth, and nose emerge as the principal focal points of interest. As the number of selected features increases, the distribution of these features progressively encompasses the forehead, eyes, eyebrows, nose, mouth, and cheeks, aligning with the key areas recognized in human vision during face recognition tasks. This means that FWFGFS can effectively identify key discriminative features and perform feature selection in a reasonable manner.

### 4.8. T-SNE Visualization Experiment

**Figure 9:**
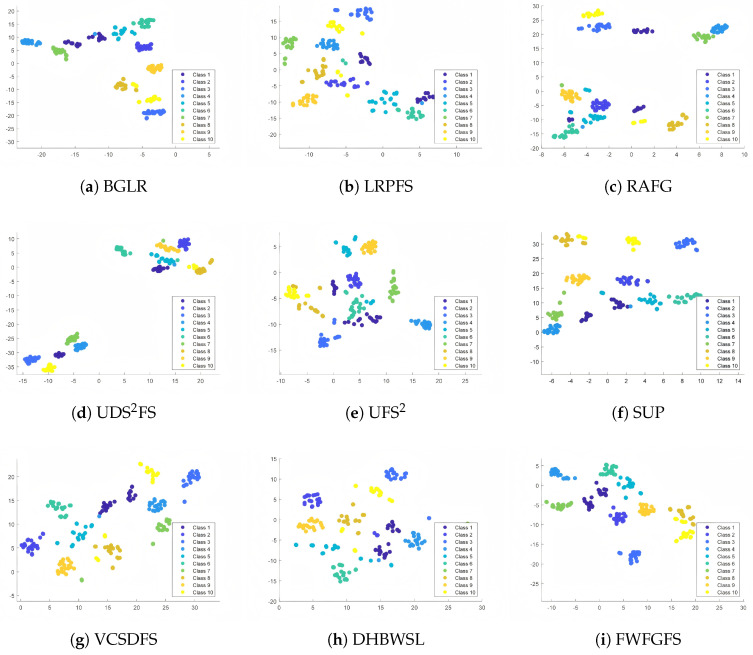
T-SNE visualization on Jaffe dataset.

Mapping high-dimensional data to a two-dimensional subspace is one of the most intuitive ways to evaluate the quality of a low-dimensional subspace. Therefore, we use t-SNE to visualize the low-dimensional data learned from the Jaffe dataset. To ensure fairness, all algorithms are used to select 100 features, and then t-SNE is applied to map these data into a two-dimensional scatter plot for visualization. Figure 9 shows the two-dimensional scatter plots of the low-dimensional data obtained by different algorithms.

An excellent algorithm should exhibit good inter-class separability and clear class boundaries when visualized with t-SNE. As shown in Figure 9, the performance of FWFGFS is clearly superior to that of the other comparison algorithms. Specifically, the scatter plot of FWFGFS shows that, except for a slight overlap between two samples, the inter-class distance for most of the samples is more distinct and easily separable from class boundaries since class boundaries are clear and intra-class distances are relatively small. In stark contrast, UFS^2^, LRPFS, RAFG, and UDS^2^FS exhibit inferior performance, characterized by vaguer class boundaries and considerable overlap among categories, especially in the interstices where different classifications converge.

It can also be observed that although SUP and VCSDFS attain a certain level of separation, there still exist substantial intra-class distances and misclassifications; for example, in VCSDFS, samples from classes “10” and “8” are confused, which greatly undermines their effectiveness. Among all these comparative algorithms, DHBWSL and BGLR demonstrate superior performance, albeit with a limited number of misclassified instances. For instance, DHBWSL reveals an indistinct boundary between classes “1” and “3”, resulting in several samples from class “10” being erroneously classified as belonging to class “8”. The comprehensive experimental results indicate that FWFGFS demonstrates a pronounced discriminative capability for handling large sample sizes.

### 4.9. Calculation Time Analysis

In this section, for evaluating the efficiency of our proposed method, we calculate the time cost on eight datasets, including 12 real-world datasets.

**Figure 10:**
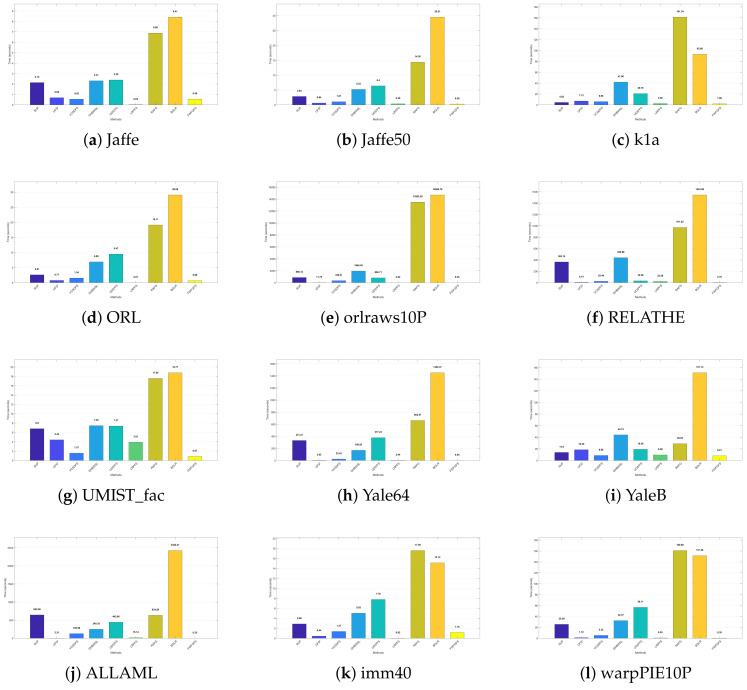
Visualization of calculation time (s) for algorithms on different datasets.

The visualization results in Figure 10 clearly demonstrate that as the dimensionality and sample size of the dataset increase, the computational time of all algorithms exhibits a consistent upward trend. This phenomenon is particularly noticeable in the orlraws10P and YaleB datasets, confirming that higher dimensionality and larger sample sizes significantly degrade runtime performance. Among the evaluated algorithms, those based on adaptive graph learning (e.g., RAFG and BGLR) generally demand substantially longer computation times. A comparative analysis reveals that FWFGFS achieves superior efficiency, with an average runtime of merely 1.50 s. In contrast, the competing algorithms—SUP, UFS^2^, VCSDFS, DHBWSL, UDS^2^FS, LRPFS, RAFG, and BGLR—exhibit significantly longer runtimes of 189.11, 4.74, 45.33, 245.95, 149.21, 4.80, 1349.79, and 1718.07 s, respectively. This stark difference highlights the computational advantage of FWFGFS over the other methods.

### 4.10. Analysis of Parameters of Fuzzy Anchor Graph

The number of anchors, neighbors, and fuzzy degrees has varying degrees of influence on the algorithm’s performance. To this end, this section provides a detailed analysis of the number of anchors, neighbors, and fuzzy degrees in fuzzy anchor graphs across six datasets.

**Figure 11:**
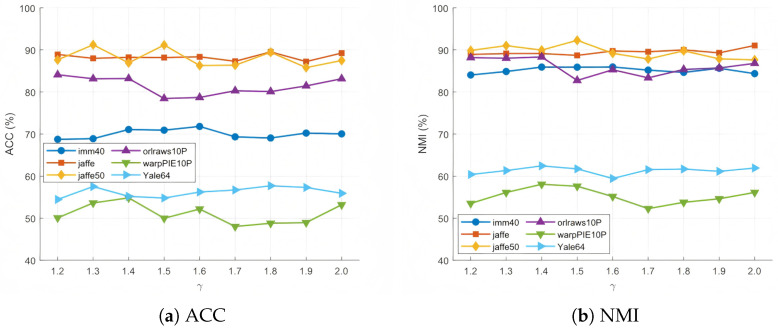
Clustering accuracy and normalized mutual information for different values of the parameter γ on six datasets.

As shown in Figure 11, the impact of fuzziness variations on algorithm performance is relatively complex. On the Jaffe50 dataset, the ACC reaches its highest value (91.22%) when the fuzzy degree is 1.3, while the NMI peaks (92.28%) at a fuzzy degree of 1.5, indicating that a moderate level of fuzzy degree can effectively balance the flexibility and accuracy of membership degrees. On the orlraws10P dataset, both ACC and NMI perform best (84.10% and 88.33%, respectively) when the fuzzy degree is 1.2, but further increases in fuzzy degree lead to performance degradation, suggesting that this dataset is more sensitive to fuzzy degree selection. Overall, when the fuzzy degree ranges between 1.3 and 1.6, most datasets exhibit superior performance, demonstrating that an intermediate level of fuzzy degree can better model data uncertainty.

**Figure 12:**
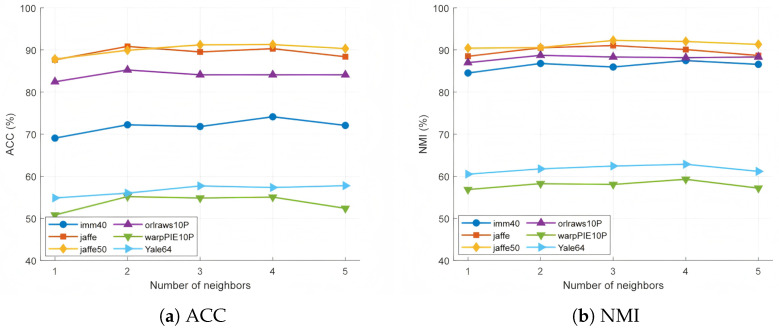
Clustering accuracy and normalized mutual information for different numbers of neighbors on six datasets.

As illustrated in Figure 12, increasing the number of neighbors significantly aids in improving performance across datasets. For example, on the imm40 dataset, when the number of neighbors is 7, the ACC reaches 74.13%, and the NMI reaches 87.48%, significantly higher than the results with only one neighbor. Similarly, on the Jaffe50 dataset, both ACC and NMI achieve their best performance (91.29% and 92.02%, respectively) when the number of neighbors is 7. However, on the warpPIE10P dataset, the performance arrives at its peak when the number of neighbors is 5. This suggests that a larger number of neighbors contributes to capturing more complex local structures, but an excessively high number may introduce redundant information, thereby reducing performance.

**Figure 13:**
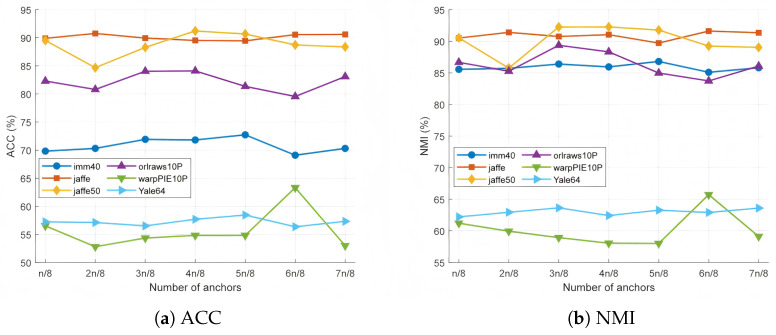
Clustering accuracy and normalized mutual information for different numbers of anchors on 6 datasets.

As also shown in Figure 13, the impact of the number of anchor points on algorithm performance varies across datasets. On the Jaffe50 dataset, when the number of anchors is $\frac{4n}{8}$, the ACC reaches its highest value (91.22%) and the NMI is 92.28%, indicating that a moderate number of anchors can better capture the local structure of the data. However, on the warpPIE10P dataset, the ACC peaks at 63.33% when the number of anchor points is $\frac{6n}{8}$, but performance fluctuates significantly with other anchor counts, suggesting that this dataset is more sensitive to the selection of anchors. Overall, when the number of anchors is $\frac{4n}{8}$ or $\frac{5n}{8}$, most datasets (such as imm40, Jaffe, and Yale64) exhibit better performance, demonstrating that a moderate number of anchors strikes a good balance between computational efficiency and model performance.

## 5. Conclusions

In this paper, the research work mainly focuses on the challenges faced by graph-based UFS algorithms in dimensionality reduction tasks; traditional graph structures focus on exploring neighborhood relationships for the global data and ignore the uncertain and fuzzy relationships between the data, such that graph learning is computationally expensive while sample points are incorrectly clustered. Moreover, the feature selection process is prone to interference by noisy features and redundant features, which affects the selection of discriminative features. Additionally, the low-dimensional representation matrix generated during the feature selection process often fails to present a clear clustering structure. Specifically, the proposed fuzzy neighborhood-based manifold learning approach is introduced, preserving local structures through fuzzy neighborhood similarity relationships between nodes while reducing the computational complexity of graph learning. Additionally, FWFGFS is constructed by designing a fuzzy weighting mechanism to adaptively adjust feature importance via the weight matrix *H*, effectively mitigating the impact of noisy samples and irrelevant features. Finally, the orthogonal tri-factorization of the low-dimensional representation matrix is utilized to extract clearer clustering structures and more independent cluster centers.

However, FWFGFS has certain limitations, particularly in parameter optimization, as it involves tuning three parameters, which increases optimization time. Future work will focus on developing adaptive parameter adjustment strategies to reduce the computational overhead of parameter tuning and further enhance the efficiency of the algorithm.

## Figures and Tables

**Table 1:** Notation description.

Notation	Description
X	Data matrix of size d×n
W	Projection matrix of size c×d
V	Clustering indicates matrix of size c×n
R	Auxiliary matrix of size c×c
U	Clustering center matrix of size c×c
S	Fuzzy anchor graph matrix of size n×n
H	Fuzzy weighting matrix of size c×n
G	Fuzzy similarity matrix of size n×n
D	Degree matrix of size n×n
L	Laplacian matrix of size n×n
I	Identity matrix of size c×c
${∥\cdot ∥}_{F}$	Frobenius norm of matrix
Tr(·)	Trace of matrix
⊙	Element multiplication of matrix
$1_{n}$	Vector of all ones of size n×1

**Table 2:** The detailed information of datasets.

Dataset	Size	Dimensionality	Class	Type
ORL	400	1024	40	Face image
YaleB	2414	1024	38	Face image
imm40	240	1024	40	Face image
ALLAML	72	7219	2	Biological
warpPIE10P	210	2420	10	Face image
Jaffe	213	676	10	Face image
orlraws10P	100	10,304	10	Face image
RELATHE	1427	4322	2	Text
Jaffe50	213	1024	10	Face image
Yale64	165	4096	15	Face image
UMIST_fac	575	1024	20	Face image
kla	2340	1326	6	Text

**Table 3:** Clustering accuracy (ACC ± STD%) of 9 methods on 12 datasets.

Datasets	SUP	UFS^2^	VCSDFS	DHBWSL	UDS^2^FS	LRPFS	RAFG	BGLR	FWFGFS
ORL	52.98 ± 2.69	41.07 ± 1.67	50.53 ± 2.11	56.05 ± 2.45	50.55 ± 2.20	47.57 ± 2.38	54.43 ± 2.16	53.55 ± 2.09	**56.37 ± 3.00**
	(100)	(100)	(60)	(100)	(30)	(90)	(100)	(100)	**(100)**
YaleB	12.58 ± 0.48	22.36 ± 1.28	10.03 ± 0.34	17.56 ± 0.35	10.39 ± 0.58	16.21 ± 0.37	9.41 ± 0.21	13.09 ± 0.45	**22.62 ± 0.98**
	(30)	(80)	(50)	(20)	(30)	(20)	(40)	(20)	**(40)**
imm40	57.35 ± 2.61	55.45 ± 2.34	45.68 ± 2.53	53.83 ± 2.02	52.27 ± 2.08	60.29 ± 3.25	59.60 ± 3.41	55.18 ± 3.05	**71.81 ± 2.61**
	(70)	(80)	(100)	(90)	(100)	(40)	(20)	(70)	**(30)**
ALLAML	70.34 ± 0.81	71.92 ± 0.02	85.69 ± 0.12	89.35 ± 0.94	78.37 ± 0.75	76.11 ± 2.14	74.65 ± 1.85	74.79 ± 0.51	**90.62 ± 1.95**
	(30)	(20)	(30)	(20)	(20)	(100)	(100)	(90)	**(20)**
warpPIE10P	26.61 ± 1.17	50.69 ± 3.04	28.16 ± 1.67	42.11 ± 2.98	37.97 ± 2.57	33.88 ± 1.83	52.50 ± 2.43	26.95 ± 1.55	**54.83 ± 2.58**
	(60)	(50)	(60)	(90)	(90)	(20)	(20)	(30)	**(40)**
Jaffe	86.03 ± 5.09	76.12 ± 6.61	83.00 ± 4.15	89.41 ± 4.18	84.69 ± 5.51	80.02 ± 5.86	85.39 ± 4.01	88.94 ± 6.23	**89.53 ± 5.45**
	(70)	(100)	(80)	(60)	(20)	(100)	(100)	(90)	**(70)**
orlraws10P	76.45 ± 4.53	55.70 ± 2.40	66.85 ± 4.90	82.45 ± 4.33	67.90 ± 5.34	67.65 ± 4.59	80.30 ± 4.02	75.75 ± 4.71	**84.10 ± 4.19**
	(20)	(100)	(90)	(50)	(20)	(60)	(90)	(100)	**(90)**
RELATHE	54.66 ± 0.02	54.75 ± 0.18	54.65 ± 0.03	59.55 ± 0.12	59.00 ± 0.02	59.09 ± 0.05	55.18 ± 0.49	54.66 ± 0.14	**59.67 ± 1.12**
	(100)	(60)	(100)	(70)	(50)	(60)	(80)	(100)	**(30)**
Jaffe50	81.97 ± 5.22	62.74 ± 3.10	73.23 ± 3.57	79.92 ± 4.48	77.93 ± 6.42	81.50 ± 2.76	84.64 ± 5.12	82.69 ± 4.03	**91.22 ± 5.20**
	(100)	(100)	(50)	(100)	(80)	(100)	(100)	(100)	**(100)**
Yale64	52.66 ± 3.31	41.09 ± 3.08	46.39 ± 1.97	44.96 ± 3.91	47.51 ± 2.85	41.15 ± 1.73	55.00 ± 4.30	52.21 ± 3.00	**57.69 ± 3.23**
	(90)	(90)	(30)	(100)	(20)	(80)	(100)	(90)	**(90)**
UMIST_fac	45.67 ± 2.24	47.65 ± 2.05	46.16 ± 2.15	47.98 ± 3.59	48.20 ± 2.14	50.15 ± 3.02	51.24 ± 3.41	45.36 ± 1.73	**51.87 ± 3.15**
	(100)	(50)	(90)	(60)	(40)	(100)	(50)	(40)	**(60)**
k1a	32.81 ± 2.01	59.31 ± 0.01	33.88 ± 0.38	42.66 ± 0.29	38.26 ± 0.49	59.35 ± 0.23	59.18 ± 0.02	34.65 ± 1.53	**59.35 ± 0.02**
	(70)	(20)	(40)	(70)	(20)	(30)	(20)	(20)	**(30)**

**Table 4:** Normalized Mutual Information (NMI ± STD%) of 9 methods on 12 datasets.

Datasets	SUP	UFS^2^	VCSDFS	DHBWSL	UDS^2^FS	LRPFS	RAFG	BGLR	FWFGFS
ORL	73.27 ± 1.60	62.50 ± 0.97	70.95 ± 1.06	74.78 ± 1.21	70.40 ± 1.41	69.05 ± 1.28	73.49 ± 1.26	73.07 ± 1.33	**74.87 ± 1.49**
	(100)	(100)	(60)	(40)	(30)	(90)	(90)	(100)	**(100)**
YaleB	20.57 ± 0.66	**36.17 ± 0.54**	14.48 ± 0.54	28.13 ± 0.48	16.22 ± 0.81	25.99 ± 0.26	15.01 ± 0.23	22.55 ± 0.49	33.26 ± 0.62
	(30)	**(80)**	(50)	(20)	(20)	(20)	(40)	(20)	(40)
imm40	77.31 ± 1.27	75.68 ± 1.47	68.70 ± 1.27	74.22 ± 1.18	72.84 ± 1.15	78.40 ± 1.36	78.34 ± 1.18	74.74 ± 1.48	**85.95 ± 1.07**
	(70)	(70)	(80)	(30)	(100)	(40)	(40)	(20)	**(30)**
ALLAML	12.51 ± 0.99	11.23 ± 2.53	37.86 ± 2.55	47.92 ± 3.88	15.58 ± 0.88	18.42 ± 2.75	16.76 ± 4.33	16.52 ± 0.62	**53.51 ± 2.41**
	(30)	(90)	(30)	(20)	(20)	(100)	(20)	(90)	**(30)**
warpPIE10P	26.17 ± 1.93	54.73 ± 1.95	25.19 ± 1.66	45.49 ± 3.03	41.81 ± 2.21	26.29 ± 1.94	55.13 ± 1.48	26.45 ± 2.29	**58.06 ± 2.36**
	(60)	(70)	(60)	(90)	(90)	(30)	(50)	(40)	**(40)**
Jaffe	89.04 ± 2.77	78.59 ± 3.66	83.84 ± 2.29	90.32 ± 2.54	87.80 ± 2.92	82.24 ± 3.90	87.89 ± 1.91	90.70 ± 3.68	**91.05 ± 2.93**
	(70)	(100)	(80)	(70)	(20)	(100)	(30)	(90)	**(30)**
orlraws10P	80.22 ± 1.82	64.69 ± 1.69	70.64 ± 3.23	85.46 ± 2.34	73.31 ± 4.16	69.12 ± 2.09	82.51 ± 2.48	80.48 ± 2.73	**88.32 ± 2.74**
	(20)	(100)	(90)	(50)	(20)	(100)	(90)	(100)	**(90)**
RELATHE	0.08 ± 0.02	0.33 ± 0.14	0.08 ± 0.02	7.04 ± 1.72	2.19 ± 0.21	5.47 ± 0.06	0.64 ± 0.03	0.27 ± 0.13	**7.07 ± 0.32**
	(100)	(80)	(100)	(60)	(50)	(20)	(30)	(30)	**(100)**
Jaffe50	82.59 ± 2.81	70.06 ± 2.65	71.33 ± 2.56	79.51 ± 2.66	83.22 ± 3.52	83.20 ± 2.15	84.77 ± 2.65	82.57 ± 2.32	**92.27 ± 3.32**
	(40)	(100)	(50)	(100)	(80)	(100)	(90)	(40)	**(80)**
Yale64	57.54 ± 2.43	47.33 ± 2.64	50.07 ± 1.44	50.58 ± 3.02	54.56 ± 1.68	46.07 ± 2.18	60.22 ± 2.86	56.05 ± 2.79	**62.43 ± 1.98**
	(90)	(90)	(30)	(100)	(20)	(80)	(100)	(90)	**(100)**
UMIST_fac	65.19 ± 1.62	62.65 ± 1.50	63.51 ± 1.64	67.23 ± 2.28	63.03 ± 1.62	66.55 ± 1.28	69.39 ± 1.44	64.54 ± 1.69	**69.87 ± 1.26**
	(70)	(80)	(90)	(60)	(70)	(100)	(80)	(60)	**(60)**
k1a	8.45 ± 0.24	0.32 ± 0.22	8.00 ± 0.06	**8.77 ± 0.42**	8.10 ± 0.09	1.97 ± 0.31	1.88 ± 0.25	8.28 ± 0.28	1.04 ± 0.38
	(70)	(80)	(40)	**(70)**	(20)	(40)	(20)	(20)	(90)

**Table 5:** Description of noisy datasets.

Datasets	Original Dataset	Data Type	Noise Type and Level
OR_8	ORL	Face image	Blocknoise (8×8)
OR_12	ORL	Face image	Blocknoise (12×12)
OR_16	ORL	Face image	Blocknoise (16×16)
imm40_8	imm40	Face image	Blocknoise (8×8)
imm40_12	imm40	Face image	Blocknoise (12×12)
imm40_16	imm40	Face image	Blocknoise (16×16)

**Table 6:** Clustering accuracy (ACC ± STD%) of 9 algorithms on 6 noisy datasets.

Datasets	SUP	UFS^2^	VCSDFS	DHBWSL	UDS^2^FS	LRFGS	RAFG	BGLR	FWFGFS
OR_8	52.45 ±	40.67 ±	49.90 ±	50.97 ±	49.80 ±	48.26 ±	53.38 ±	52.31 ±	**55.15 ±**
	2.62 (100)	1.40 (100)	2.11 (60)	2.08 (80)	2.15 (40)	2.80 (100)	1.96 (100)	2.83 (100)	**2.07 (60)**
OR_12	51.58 ±	40.52 ±	49.30 ±	50.75 ±	48.00 ±	47.40 ±	53.28 ±	52.21 ±	**53.43 ±**
	3.15 (100)	1.26 (100)	2.34 (100)	2.91 (100)	1.72 (40)	2.28 (100)	2.56 (90)	2.19 (100)	**2.88 (100)**
OR_16	51.28 ±	40.76 ±	49.20 ±	50.38 ±	47.71 ±	45.62 ±	53.38 ±	51.78 ±	**53.95 ±**
	2.54 (100)	2.15 (100)	1.83 (100)	2.33 (80)	2.03 (50)	2.04 (100)	2.87 (90)	2.02 (100)	**2.12 (60)**
imm40_8	66.77 ±	53.54 ±	43.27 ±	50.77 ±	57.31 ±	59.50 ±	59.87 ±	51.45 ±	**70.14 ±**
	3.14 (90)	2.75 (70)	2.11 (20)	3.08 (90)	2.95 (100)	3.29 (40)	3.39 (60)	2.46 (60)	**3.84 (20)**
imm40_12	57.12 ±	53.14 ±	40.14 ±	52.37 ±	57.37 ±	55.41 ±	63.60 ±	54.18 ±	**68.18 ±**
	2.48 (60)	2.36 (100)	2.09 (20)	2.03 (30)	1.73 (90)	3.42 (100)	2.84 (20)	2.34 (50)	**3.00 (20)**
imm40_16	55.66 ±	53.14 ±	38.85 ±	50.89 ±	47.06 ±	50.83 ±	59.54 ±	52.04 ±	**69.66 ±**
	2.89 (50)	2.33 (20)	1.92 (100)	3.26 (30)	2.63 (20)	2.02 (100)	3.42 (70)	3.10 (50)	**2.81 (20)**

**Table 7:** Normalized Mutual Information (NMI±STD%) of 9 algorithms on 6 noisy datasets.

Datasets	SUP	UFS^2^	VCSDFS	DHBWSL	UDS^2^FS	LRFGS	RAFG	BGLR	FWFGFS
OR_8	72.48 ±	62.08 ±	69.93 ±	71.84 ±	69.67 ±	68.52 ±	72.76 ±	72.21 ±	**73.41 ±**
	1.52 (100)	1.31 (100)	1.37 (100)	1.31 (80)	1.17 (30)	1.55 (100)	1.30 (100)	1.74 (100)	**1.19 (70)**
OR_12	71.94 ±	61.47 ±	69.23 ±	70.81 ±	68.10 ±	67.62 ±	72.79 ±	71.81 ±	**72.80 ±**
	1.69 (100)	0.91 (100)	1.38 (100)	1.56 (80)	1.14 (40)	1.37 (100)	1.41 (90)	1.60 (100)	**1.75 (80)**
OR_16	71.13 ±	61.18 ±	69.90 ±	70.50 ±	68.41 ±	68.75 ±	72.56 ±	71.43 ±	**72.74 ±**
	1.65 (100)	1.02 (100)	1.59 (100)	1.48 (80)	1.17 (30)	1.35 (100)	1.23 (100)	1.15 (100)	**1.27 (80)**
imm40_8	82.75 ±	73.87 ±	66.68 ±	72.61 ±	77.31 ±	78.30 ±	78.51 ±	72.79 ±	**85.76 ±**
	1.43 (90)	1.58 (70)	1.66 (100)	1.22 (30)	1.48 (100)	1.62 (40)	1.19 (40)	1.01 (60)	**1.42 (30)**
imm40_12	77.99 ±	74.21 ±	64.25 ±	73.92 ±	77.15 ±	74.38 ±	80.27 ±	74.06 ±	**83.47 ±**
	1.13 (60)	1.14 (90)	1.20 (20)	1.21 (30)	1.45 (100)	1.87 (100)	1.43 (20)	1.29 (50)	**1.36 (20)**
imm40_16	77.89 ±	73.42 ±	63.10 ±	73.04 ±	69.90 ±	70.78 ±	78.33 ±	72.72 ±	**84.76 ±**
	1.03 (50)	1.05 (20)	1.50 (100)	1.46 (30)	1.33 (20)	1.41 (100)	1.30 (40)	1.37 (50)	**1.26 (20)**

## Data Availability

The original datasets presented in the study are openly available at https://jundongl.github.io/scikit-feature/datasets.html and https://www.face-rec.org/databases/ (accessed on 28 July 2025). The detailed experimental data presented in this study are available on request from the corresponding author due to ongoing research efforts.
